# Learning Design for Future Higher Education – Insights From the Time of COVID-19

**DOI:** 10.3389/fpsyg.2021.647948

**Published:** 2021-07-15

**Authors:** Daniela Dumulescu, Irina Pop-Păcurar, Constantin Valer Necula

**Affiliations:** ^1^Faculty of Psychology and Education Sciences, Babeş-Bolyai University, Cluj-Napoca, Romania; ^2^“Andrei şaguna” Faculty of Orthodox Theology, Lucian Blaga University, Sibiu, Romania

**Keywords:** teaching and learning design, higher education, psychology-based instructional design, micro-course design, autonomy-focused teaching strategies, COVID-19

## Abstract

The COVID-19 pandemic brought many challenges in higher education. All teaching and learning activities were moved online. Universities had to provide adapted solutions to facilitate learning and maintain students’ engagement. Online education implies creating new learning environments with the help of digital technologies. Beyond the process of acquisition of knowledge, teachers needed to facilitate cooperative learning, build positive relations, and reduce negative emotions. We provide some expert insights based on empirical observations on teaching and assessment practices connected with psychology models applied in education. The aim of the paper is to formulate specific learning design recommendations for developing effective didactic strategies and addressing the current worldwide critical issue: dealing with digitization of higher education in the immediate future. We propose a model of university classes aimed at bringing together our experience as teachers of psychology and didactics with evidence-based cognitive-educational theories and practices. The result is an example of an instructional work-model based on the complex dynamic between cognitive, emotional-motivational, and social aspects of learning in online settings. The effectiveness of university teaching in the post-digital era is strongly connected with the ability to create cognitive-transferable learning experiences, emotionally safe learning environments, while promoting an active autonomy-focused approach for self-regulated learning.

## Introduction

Education is always affected by the context in which it is enacted. From local specificity to regional and global perspectives, 2020s COVID-19 pandemic profoundly impacted individuals and communities all over the world. Some trends in attitudes and behaviors which emerged in the last years became the new realities: a growth in the use of social media, more cultural and social gaps, a rise in anti-intellectualism in public life, changes in educational patterns, climate change, new labor market disparities, digitization etc. (Madalinska-Michalak et al., 2018).

COVID-19 pandemic has launched a digital revolution in higher education, bringing a lot of important changes in a very short time (Strielkowski and Wang, 2020). Digital tools appeared as a universal solution for education, ready to welcome cohorts of students and teachers. Therefore, an interesting issue is how universities will respond to this ongoing change of beneficiaries’ realities.

Helping students to develop the skills and attitudes they need to provide innovative responses to the changing world and the demands of their future employers became a strong requirement. The effectiveness of university teaching in the post-digital era is strongly connected with the ability to create cognitive-transferable learning experiences, emotionally safe learning environments, and promoting an autonomy-focused approach for self-regulated learning.

Engaged in the most recent context, this paper focuses on new insights into the challenges facing the academic environment since almost all teaching and learning activities were moved online. Since online education implies a different approach, related to creating new learning environments combining digital technologies and adapting the most recent findings in psychology, it is useful to ask what professional roles and core practices should be reconsidered? and how can university teachers facilitate cooperative learning, build positive relations, and an emotionally safe online learning environment? Building on the authors’ personal insights, empirical observations, and practice, the present paper illustrates some examples of online teaching design activities responding to current tensions, changes and students’ expectations, and emerging needs.

## Addressing the Problem: Redesigning Teaching and Learning in Higher Education in Online Settings

The challenges which the COVID-19 pandemic brought in academia triggered the need to find answers to many existing reflective and research questions concerning the specificity of online education. The following questions remain, and challenge educators who are interested in various types of evidence-based instructional solutions for online settings:

### Which Aspects Should Be Considered While Designing an Online/Virtual Learning Environment?

Based on empirical research data published at the very beginning of computer-based learning, Nistor (2003) noticed the problems refer mainly to the social context of learning. Moreover, learners are often overwhelmed by the amount of information available in the learning context, by the unusual learning approach, which leads to inefficient processing of information and even to quit the learning process. To avoid these problems, additional support is necessary. But what should this support look like?

Learning designers should build online learning environments and courses which emphasize cognitive, emotional, motivational, and social aspects of learning. Evidence-based theories in psychology, such as (but not limited to) Cognitive Load Theory, Self-Determination Theory (Ryan and Deci, 2017), Knowledge Learning Framework (Koedinger et al., 2012), Cognitive Theory of Multimedia Learning (Mayer, 2019), Situativity Theory (Barab and Duffy, 2000), Value Theory of Achievement Emotion (Pekrun, 2000), Constructivist Theory (Airasian and Walsh, 1997), and Social Cognitive Theory of Learning (Bandura, 1997), should also be included. We argue the need for designing online courses that will increase the participation and wellbeing of students. Some key aspects derived from evidence-based theories mention above should be related to the learning activity is triggered by a problem that is relevant for the students; learning should take place in a social context; learning activities should include various active teaching and learning methods; the key attribute for the learner is motivation; there is a reciprocal relation between cognition and emotion when the learner is organizing and operates with information; and the instructional support in virtual environments is essential in self-regulation of learning. At this point, another critical issue arises are the university teachers sufficiently trained and committed to offer effective instructional support to students online?

### What Is and What Is Not an Online Course? What Are the Criteria, If Any?

At the beginning of 2020, when teachers and students moved their collective efforts online, PowerPoint presentations first migrated as they were, from amphitheaters and laboratories, *via* screen-sharing in online meetings hosted by different platforms. The main problem is that PowerPoint slides on the Internet are pieces of information, not a course. An online course tends to be a complex, structured and rich learning environment, carefully developed by a team of highly qualified experts on the subject content, pedagogy, and ICT (Bjørke, 2017). Courses designed for online learning environments are advanced, well structured, and interactive. They are led or guided by a teacher or tutor (tutor-guided courses), giving advice, motivating, assisting groups, etc.

The *flipped classroom approach* mixing synchronous and asynchronous tasks and activities (Brame, 2013; Bjørke, 2017) grows into an interesting topic for university teachers during the recent active-learning webinars. Students access content and engage in activities designed to develop their understanding before class, and then, use the course time to discuss and engage in-depth with issues, ideas, and questions arising from the pre-course content and activities (Farmer, 2015). Using flipped learning in higher education enhances critical thinking skills, self-learning, experience building, and communication and collaboration skills among students (Mahasneh, 2020) but requires more attention to students’ activity guidelines. Guided by less to more complex tasks, students autonomously perform the lower levels of cognitive work and focus on the higher forms of cognitive work, supported by their peers and by the professor. Virtual learning environments require understanding the learner’s profiles and using cognitive, emotional, behavioral, and motivational information, to negotiate meaning and solve problems in collaborative and constructive ways (Bjørke, 2017).

### How and When Students Are Learning the Critical Skills Associated With e-Learning?

Significant studies show that most students’ information processing skills are haphazardly caught rather than specifically learned (Chappell, 2018). This phenomenon occurs even more frequently in online courses, even for students with strong e-learning skills (Chappell, 2018). Course content that requires multiple perspectives, and controversial issues are useful for creating one of these activities. Chappell (2018) also presents a few steps for designing critical information processing activities: (1) the theory or concept used; (2) the teacher should provide digital resources to show multiple angles, perspectives, or ideas; (3) the teacher and students should ask simple questions that require analysis, research, and evaluation to answer; and (4) the teacher should consider that critical information processing skills are best taught rather than caught.

### What Are the Elements That Can Make (Blended and) Online Learning Successful?

Blended and online courses redefine traditional educational roles and provide different opportunities for learning. According to Smith and Brame (2013), there are some critical aspects of effective online education. Firstly, acknowledging what students bring to the online classroom (background, needs, and interests) and what they take away as relevant and meaningful outcomes is essential. Secondly, including collaborative discussions and small group assignments creates a “level playing field” for disadvantaged students. Thirdly, it is important that students understand which behaviors help them learn and proactively apply those strategies. This awareness and knowledge of one’s personal learning process involve increased metacognition – a key practice for students’ self-regulated learning. Fourthly, by self-monitoring their time and pacing, students can control their learning and are able to spend more time on unfamiliar or difficult content. Fifthly, the immediate feedback provided in multiple manners in online settings is a very useful aspect of online learning. It is easier for learners to contact instructors or peers *via* email/learning platform/chat. In addition, online tests and quizzes can be constructed with automatic grading capability that provides timely feedback (Smith and Brame, 2013). Last but not least, using multimodal materials can be used to increase engagement, autonomy, and self-regulation.

## Guidelines for Course Design in Online Settings

Our proposed instructional work-model is taking into consideration both internal factors (cognitive processes) and external contextual variables and the reciprocal relation between them, promoting a co-constructive process of meaning and facilitating higher-order learning outcomes (Mayer, 2019). Moreover, our approach in proposing the current guidance instruction model is based on the urgent need for flexible online learning courses, adapted and personalized to cognitive, emotional, motivational, and social needs (Redding, 2014). Enhancing students wellbeing and satisfaction related to learning should be a priority goal for higher-education professionals in a post-pandemic world.

**Figure 1:**
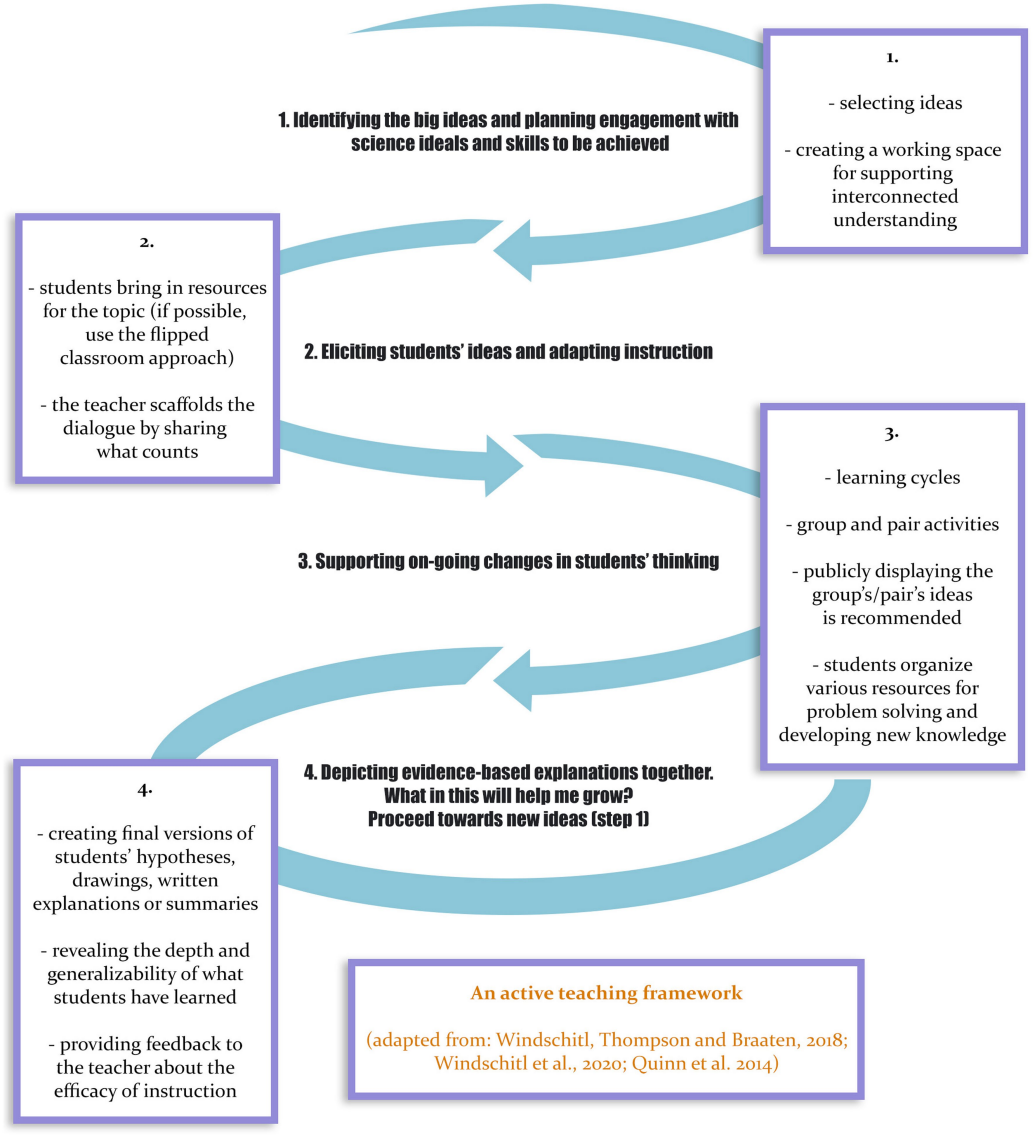
An active teaching framework.

Bringing together the theoretical and methodological assertions presented in the previous sections, we propose a general frame for designing active online/blended courses, with four stages (adapted from Quinn et al., 2014; Windschitl et al., 2018, 2020). They are different facets of effective practice that can be connected for promoting successful teaching actions (Figure 1):

1. *Identifying the main ideas and planning engagement with science information and skills to be achieved* – it requires the teacher to select the relevant ideas for the students in constructing new knowledge or skills. The course must connect individual learning experiences and interconnected understandings.
2. *Eliciting students’ ideas and adapting instruction* – teacher figure out the resources students bring to the topic (possible, flipped classroom approach). Sensemaking discourse of students is essential in this state. The teacher is scaffolding the dialogue by sharing the main ideas. Explicit thinking allows students to learn through modeling.
3. *Supporting ongoing changes in students’ thinking* – it involves cycles of learning activities based on learning support materials and interactive direct (synchronous) instruction. Group/pair activities, the group’s ideas presentation is recommended. Students learn to organize various resources for solving problems and developing new knowledge. Peer/group presentations also support the development of students’ academic discourse.
4. *Depiction together with evidence-based explanations*. *What is in here for my growth?* – proceed toward new ideas (to step 1) – supports students in using ideas from the previous activities to revise their current explanations and scientific models. Students (individual and pairs) are asked to make final versions of their hypothesis, explanations, summaries, and to examine records of their thinking from earlier in the course/laboratory. This step reveals the depth and generalizability of what student has learned. At the same time, provides feedback to the teacher about the efficacy of instruction, including tools and routines that need to be modified.

Taking the case of science teaching and learning course (pre-service teacher education, fourth semester), we create the opportunity for knowledge to be constructed by the students, using a problem-based learning approach, which was previously associated with greater relatedness, autonomy, and control, but also with an increase of intrinsic motivation (Wijnen et al., 2018), reduced students stress (Moffat et al., 2004), retention of information, and critical thinking skills (Schwartz et al., 2001). The activity is online managed using a platform that features the option of breakout rooms/channels (e.g., Microsoft Teams, Zoom, and Google Meet). The teaching and learning strategies include cooperative learning design adapted for higher education (Ertl and Stegmann, 1999), the ground rules of the flipped classroom (Farmer, 2015; Mahasneh, 2020), and project-based learning (do Amaral et al., 2015; Ulrich, 2016) which are evaluated through different tasks, assignments, time, and material management (implementation of general frame stages 1, 2, 3, and 4 mentioned above). The professor may provide students with guidelines for Socio-Scientific Inquiry-Based Learning (Amos et al., 2020); successful promotion of engaged learning is particularly reported when the inquiry questions involving local and personal relevance (implementation of general frame stages 3 and 4 mentioned above). The key problem presented to the students is to produce a learning environment needed for teaching biology lessons online as well. We divided the problem into sub-tasks: (1) acquiring information on the Internet and evaluating learning materials and online learning environments; (2) strategic processing of information and preparing materials for teaching specific biology lessons. Scientific content selection is, by choice, from the Biology National Curriculum for the secondary or high school levels (mainly related to the general frame stages 2, 3, and 4).

During the problem-based assignment, students need to be active, to work together, build a biology lesson, a methodological frame, an online-appropriate environment for this lesson, and then to present the “product” to the other groups (mainly related to the general frame stages 2 and 4).

The whole course includes lesson presentations (micro-teaching, role play, and mentoring), but nevertheless, it is designed on-site or online (mainly related to the general frame stages 3 and 4). Even if the content – teaching biology in an online environment – is pre-specified, each group can decide which aspect of the subject they want to work on and which resources they want to use. Also, students can decide when to work together or alone, dividing the task and which kind of activity fits their learning style (Ertl and Stegmann, 1999). There is a meeting/lesson/course every week and a deadline for every task, but there is also a time for incubating ideas and personalizing the learning process and outcomes (mainly related to the general frame stages 2 and 4). The tools students use and the current problems they deal with are strongly connected to their professional field. Also, students learn multiple perspectives of the same subject and need to build valuable and useful support resources (implementation of general frame stages 3 and 4 mentioned above, with a cycle perspective, returning to phases 1 and 2). This example demonstrates that learning – even in online settings – could be planned as an active, constructive, emotionally safe, and self-regulated process in which students autonomously create new knowledge structures and link them with the existing ones.

## Discussions

The integrated work-model, we designed as an example of online learning method is based on the complex dynamic and constant interaction between cognitive, emotional-motivational, and social aspects of learning, which are responsible for academic performance, especially in online settings and pandemic stressful context (Hendrie and Bastacini, 2020). We do not seek to propose an exhaustive and extensive model of instructional design but to offer a guideline for higher-education professionals for structuring and adapting their own courses to student’s needs. The main purpose of our article was to open reflective questions and perspectives for paying more attention to learning specificity and translating active instructional methods into e-methods and contents validated by psychological models and frameworks.

More specific, the **cognitive** aspect of learning is represented in our model by emphasizing the processes of reflective thinking, associations, and sensemaking, which is in line with the previous instructional strategies derived from the Knowledge Learning Framework theory (Koedinger et al., 2012). Moreover, the Cognitive Theory of Multimedia Learning states the importance of engaging students in appropriate active processing through selecting the relevant information, organizing, and integrating it, due to its value for promoting meaningful learning and long-term retention. At the same time, active learning in academic tasks increases in-depth learning, proved to be essential for generalization and information transfer, which is strongly related to our proposed work-model (Mayer, 2019).

**Motivational** aspects of learning are critical to learning environments which require a high level of engagement (Fiorella and Mayer, 2015; Buhr et al., 2019). Through the activities we proposed in our instructional design model, we paid greater attention to the self-efficacy cognitions (Bandura, 1997) and the three psychological needs, derived from Self Determination Theory – autonomy, control, and relatedness (Ryan and Deci, 2017). Recent research showed the significant positive effect of online courses meeting those needs on student’s psychological engagement in online learning (Huang and Mayer, 2016; Sun et al., 2019). More, a flipped classroom approach and an educational environment rich in resources for learning, positive relations, feedback, and learning goals, were proved to offer opportunities for accomplishing students psychological needs (Muir, 2020). The active roles and responsibilities of students, helps them feel connected and cognitively engaged and increase self-efficacy, which can lead to better engagement and performance in learning tasks (Bandura, 1997). Allowing students to manage and moderate their discussion in small groups increase self-efficacy, and autonomy. Mayer (2019) argues in favor of designing learning activities that provide opportunities for engaging reflection and collaboration, which is also the case of our example.

Another important dimension of learning is the **emotional** one. Promoting students’ positive and healthy emotions were essential when proposing the course design guidance. Previous studies showed that engaging in real-life problem-based activities, cultivating relations through learning, and feedback significantly improved students’ enjoyment and satisfaction in the online environment (Guo et al., 2018). The collaborative and active approach may also reduce anxiety, increasing student’s wellbeing, and reducing emotional distress (Mayer, 2019).

Last, but not least, the **social** component of learning is essential for increasing learning effectivity through human connection. From a Situativity theory (Barab and Duffy, 2000), meaningful learning is being socially constructed. A strong argument for instructional methods based on collaboration, human connection, and reflection is that it increases meaningful learning outcomes. In our model, those aspects are enhanced by working collaboratively, feedback, guidance, and modeling, which are also conditions for critical thinking and self-directed learning competencies (Mayer, 2019).

The main limitation of our proposed work-model is that it is not empirically validated; it is rather based on translating some evidence-based principles into practice, together with professional reflections and experiences. Second, even if we managed to integrate into our model all the relevant dimensions of learning and to relate them with the previous results in the field, there are still other contextual and individual factors that can interfere with the effectiveness of the instructional design. Third, a possible limitation can be related to the teacher and students’ technical skills and access to technology. Future studies should empirically address these issues.

The most important implication of our work is related to building more effective course designs in higher education, which will promote highly autonomous learners and increase student’s self-regulation and self-management skills in online settings (Wong et al., 2019). Our paper seeks to emphasize the crucial role of teacher’s insights and practices, in constructing adapted and individualized classes, especially in an online educational environment. We promote the active teaching strategies for online classes in order to highlight the positive implications of applying psychology-based instructional design principles to course design.

## Data Availability Statement

The original contributions presented in the study are included in the article/supplementary material, and further inquiries can be directed to the corresponding authors.

## Author Contributions

All authors listed have made a substantial, direct and intellectual contribution to the work, and approved it for publication.

### Conflict of Interest

The authors declare that the research was conducted in the absence of any commercial or financial relationships that could be construed as a potential conflict of interest.
